# Upper-Limb Cryoneurolysis for Painful Post-Stroke Spasticity in Severely Impaired Upper Limbs: A Feasibility Case Series

**DOI:** 10.3390/neurolint18050078

**Published:** 2026-04-23

**Authors:** José Alexandre Pereira, Frédéric Chantraine, Céline Schreiber, Tanja Classen, Evangelia Agneskis, Laurence Medinger, Silvia Morini, Gilles Areno, Xavier Masson, Frédéric Dierick

**Affiliations:** 1 Medical Department, Centre National de Rééducation Fonctionnelle et de Réadaptation—Rehazenter, Rue André Vésale 1, 2674 Luxembourg, Luxembourg; 2 RehaLAB, Centre National de Rééducation Fonctionnelle et de Réadaptation—Rehazenter, Rue André Vésale 1, 2674 Luxembourg, Luxembourg; 3 Physiotherapy Department, Centre National de Rééducation Fonctionnelle et de Réadaptation—Rehazenter, Rue André Vésale 1, 2674 Luxembourg, Luxembourg; 4 Occupational Therapy Department, Centre National de Rééducation Fonctionnelle et de Réadaptation—Rehazenter, Rue André Vésale 1, 2674 Luxembourg, Luxembourg; evangelia.agneskis@rehazenter.lu

**Keywords:** pilot, spasticity, cryoneurolysis, pain, stroke

## Abstract

Background: Post-stroke upper-limb spasticity can cause pain, hinder passive care, and lead to secondary musculoskeletal complications. Current minimally invasive treatments have important limitations. Cryoneurolysis, which creates a controlled cold lesion of peripheral nerves, may offer a partially reversible focal denervation alternative. Methods: We conducted a feasibility case series in the outpatient department of a rehabilitation centre. Three adults with chronic post-stroke hemiparesis and a non-functional spastic upper limb underwent ultrasound- and nerve stimulation-guided cryoneurolysis of the musculocutaneous, median, and/or ulnar nerves. All had demonstrated a positive response to diagnostic nerve blocks beforehand. Feasibility outcomes included completion of planned nerve targets, tolerability under local anesthesia, absence of serious adverse events, and completion of 6-month follow-up. Secondary outcomes were Modified Ashworth Scale (MAS), qualitatively assessed passive joint mobility (video-documented), pain measured by visual analogue scale, sensory testing, and electroneuromyography (ENMG). Results: All procedures were completed as planned. Treatment was well tolerated under local anesthesia, and no serious adverse events occurred. MAS decreased by at least 2 points in targeted patterns, with immediate improvement in passive mobility; these effects persisted at 6 months. Pain remained unchanged in two participants and improved in one. Sensory testing at 6 weeks was stable. ENMG findings were heterogeneous, including reduced ulnar sensory action potential amplitude and biceps denervation activity in one participant. Conclusions: In this small series, cryoneurolysis for post-stroke upper-limb spasticity was feasible and associated with sustained tone reduction and improved passive mobility. Larger controlled studies are required to better define safety, optimize targeting strategies, and assess patient-centred outcomes.

## 1. Introduction

Spasticity is a frequent consequence of upper motor neuron lesions, including stroke, traumatic brain injury, spinal cord injury, multiple sclerosis, and cerebral palsy [1,2]. In its strict neurophysiological definition, spasticity refers to a velocity-dependent increase in tonic stretch reflexes with exaggerated tendon jerks, reflecting increased excitability of the stretch reflex pathway [3,4,5]. In routine clinical practice, however, the term is often used more broadly to describe the positive features of the upper motor neuron syndrome, such as involuntary muscle overactivity, co-contractions, abnormal synergies and postures, spasms, clonus, and spastic dystonia [2,6,7]. Because these components may coexist and vary across patients, clarifying which manifestation is being targeted is essential when choosing and evaluating treatment.

In chronic post-stroke hemiparesis, upper-limb spasticity may contribute to rigid postures and impaired voluntary control, often in combination with sensory deficits. When left untreated, sustained muscle overactivity and immobility can promote secondary structural changes—including muscle–tendon shortening, joint stiffness, deformity—and may be associated with pain and skin/trophic complications [6,8]. Beyond impairment, spasticity-related upper-limb postures can interfere with hygiene, dressing, positioning, transfers, and caregiver assistance, and may negatively affect body image and participation [2,7]. Given the projected increase in stroke prevalence in Europe, scalable outpatient approaches that address spasticity-related disability remain highly relevant [9].

A persistent challenge is that spasticity is difficult to quantify with non-instrumental measures, and different clinical scales show notable intra- and inter-rater variability [10,11,12,13]. This limitation is amplified when multiple joints are involved and when treatment aims extend beyond tone reduction, including comfort, ease of care, prevention of secondary complications, caregiver burden, autonomy, and quality of life [14,15,16,17,18]. Therefore, spasticity assessment is generally performed through a goal-oriented, multilayered, and comprehensive approach [19,20,21,22,23,24].

Current therapeutic strategies combine non-invasive rehabilitation approaches (e.g., physiotherapy, casting, orthoses, functional electrical stimulation, pharmacological agents) with minimally invasive and invasive procedures according to severity, distribution, and reversibility requirements [25,26,27]. Reversible or partly reversible interventions acting on the stretch reflex/motor overactivity pathway include intramuscular botulinum toxin injections [28,29,30,31,32] and chemical neurolysis with alcohol or phenol [33,34,35,36]. More invasive options include neurosurgical procedures (e.g., peripheral neurotomy, selective dorsal rhizotomy, intrathecal baclofen, neuromodulation) [37,38,39,40,41,42,43], and in selected cases, orthopedic surgery for fixed contractures or deformities [42,43,44]. Despite this armamentarium, access, cost, and workflow constraints may limit availability, and there remains a need for additional outpatient options that are technically feasible and scalable.

In clinical decision-making, an important step is distinguishing the contribution of dynamic muscle overactivity from fixed structural stiffness. Diagnostic motor nerve blocks can temporarily reduce reflex-mediated resistance to passive movement, thereby helping to identify whether limited mobility is primarily due to neural overactivity versus altered passive viscoelastic properties of muscles/tendons or joint structures. In addition to supporting goal setting, diagnostic blocks can simulate the expected effect of interventions that reduce neural drive to the target muscles [45,46,47,48].

Cryoneurolysis (CNL) produces a focal, temperature-controlled lesion of a peripheral nerve using a cryoprobe, typically guided by ultrasound with optional electrical stimulation to refine targeting [49,50,51,52,53]. By inducing axonotmesis while preserving the connective-tissue scaffold of the nerve (Sunderland grade II) [54,55,56,57], CNL may allow functional recovery through axonal regeneration and has been used in interventional pain practice to provide prolonged regional analgesia [57,58,59,60,61,62,63,64,65]. There are some published preliminary applications of CNL in spasticity in humans [66,67,68,69,70,71,72,73], including a recent laboratory-verified post-stroke case reporting durable gait improvements after sequential motor-branches CNL [74], but key questions remain regarding feasibility in rehabilitation outpatient settings, tolerability, target selection (including mixed sensorimotor nerves), optimal dosing parameters (e.g., cycle number and duration), and neurophysiological safety signals.

The aim of this feasibility study was to evaluate whether ultrasound and stimulation-guided CNL of selected upper-limb peripheral nerves can be delivered in a rehabilitation outpatient setting, and to describe short- and medium-term changes in tone and ease of passive mobility, alongside pain, sensory testing, and electroneuromyography (ENMG) findings in a small post-stroke case series.

## 2. Materials and Methods

### 2.1. Study Design, Setting, and Ethics

We conducted a pilot and feasibility case series in the outpatient setting of the Centre National de Rééducation Fonctionnelle et de Réadaptation. The study was approved by the joint ethics committee of the CNRFR—Rehazenter and Hôpital Intercommunal de Steinfort (approval date: 16 July 2019) and was conducted in accordance with the Declaration of Helsinki. Written informed consent was obtained from all participants, including consent for video recordings.

This case series is reported in line with the Preferred Reporting of Case Series in Surgery (PROCESS) 2020 guideline [75]. However, this case series was not registered in a public registry because it was a local exploratory feasibility work.

### 2.2. Participants

Three post-stroke upper-limb chronic spastic hemiparetic outpatients were included. The participants’ general characteristics are listed in Table 1. Inclusion criteria were: (1) clinically stable status after stroke occurring >12 months previously; and (2) a non-functional spastic upper limb with Modified Ashworth Scale (MAS) score > 2 in the main involved muscle groups (elbow flexors, forearm pronators, wrist flexors, and finger flexors). Exclusion criteria were: (1) cognitive impairment preventing informed decision-making; (2) concomitant invasive spasticity treatment within 4 months before the procedure and until the end of follow-up; and (3) contraindications to CNL (cryoglobulinemia, paroxysmal cold hemoglobinuria, cold urticaria, Raynaud’s disease, and open/infected wounds at or near the treatment site).

Participants were recruited between August 2019 and October 2020. CNL procedures were performed between October 2019 and December 2019. Follow-up assessments were completed through 6 months post-procedure; the last follow-up visit occurred in June 2020. Participants were identified through the outpatient setting and were invited to participate if they met eligibility criteria and had goals focused on comfort and passive care of a non-functional upper limb. Cases were non-consecutive during the recruitment period. To ensure de-identification, participants are reported using coded identifiers (#1–#3), and potentially identifying details (exact dates, facial features in images/videos) were removed or obscured in published materials.

All participants presented a similar upper-limb spastic posture (shoulder internal rotation/adduction, elbow flexion, forearm pronation, wrist flexion, clenched hand), corresponding to a previously described upper-limb pattern [76].

### 2.3. Pre-Procedure Diagnostic Nerve Block (Target Confirmation)

Before CNL, an ultrasound- and nerve stimulation-guided diagnostic motor nerve block was performed for each selected target nerve. A total of 1 mL of 1% lidocaine was injected per nerve using a 50 mm insulated stimulation needle connected to a Stimuplex HNS 12 nerve stimulator (B. Braun^,^ Melsungen, Germany). Ultrasound guidance was provided using a GE Logiq S8 device and a 6–15 MHz linear probe (General Electric, Boston, MA, USA). Stimulation consisted of rectangular pulses with a duration of 1 ms and a frequency of 1 Hz. The current intensity was initially set at 2 mA and progressively decreased to determine the minimal stimulation threshold, defined as a motor response elicited at less than 0.5 mA.

The diagnostic block was used to support target selection by confirming that the limitation in passive mobility was predominantly due to neural overactivity rather than fixed rheological or structural stiffness. A block was considered positive when a clear reduction in resistance to passive movement was observed, with improved ease of passive mobilization and/or a visible gain in range within the targeted movement pattern.

### 2.4. Cryoneurolysis Procedure

**Figure 1:**
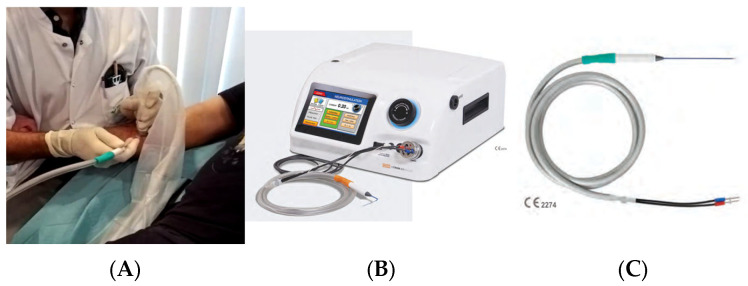
Ultrasound and neurostimulation-guided upper limb, median nerve cryoneurolysis in patient #3 (**A**) with a Metrum Cryoflex Painless-S device (**B**) using a single-use cryoprobe (**C**).

All procedures (Figure 1A) were performed under strict aseptic conditions by a single operator (J.A.P.), a physician experienced in ultrasound-guided peripheral nerve interventions. Participants were positioned supine. The treated upper limb was positioned with the shoulder in abduction and external rotation, the elbow extended, and the forearm supinated, as tolerated and as required to ensure adequate ultrasound access to the target nerves. The limb was supported to maintain stability while facilitating visualization in the axillary region (musculocutaneous nerve) and along the medial distal arm (median and ulnar nerves). Percutaneous CNL was performed using a CO_2_-based system (Metrum Cryoflex, Warsaw, Poland) (Figure 1B) under ultrasound guidance with a Logiq S8 device equipped with a 6–15 MHz linear probe.

Electrical stimulation, integrated into the Metrum Cryoflex system, was delivered at 1 Hz with a pulse duration of 0.1 ms. The current was initially set at 2 mA and gradually decreased to determine the lowest motor threshold. Adequate targeting required clear ultrasound visualization of the nerve at the tip of the probe, combined with a selective motor response—corresponding to contraction of muscles innervated respectively by the musculocutaneous, median, or ulnar nerve—elicited at ≤0.5 mA. Patient #1 did not undergo ulnar nerve CNL.

For each targeted nerve, two consecutive freezing cycles of 2.5 min were delivered, separated by a 1 min passive thaw interval. Temperatures were expected to reach approximately −72 to −75 °C at the probe tip (ice-ball core) and around −40 °C in the surrounding tissue, depending on local heat sink effects. Single-use straight cryoprobes were employed (120 mm length, 1.3 mm diameter [16G], triangular tip), Figure 1C. A 14G trocar cannula was used to guide probe insertion through the skin and subcutaneous tissue and to protect the skin during the procedure. Previously, local anesthesia with 1 mL of 1% Xylocaine was administered in the superficial fascial plane and subcutaneous tissue at the skin entry site of the trocar cannula and cryoprobe.

Participants continued usual care (physiotherapy and/or occupational therapy focused on positioning, stretching, and hygiene-related handling) during follow-up; no additional invasive spasticity procedures were performed during the study period.

### 2.5. Outcomes and Follow-Up Schedule

**Figure 2:**
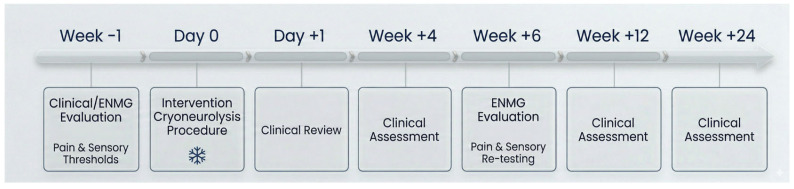
Study design timeline.

Follow-up visits and assessments were conducted in the outpatient rehabilitation setting at baseline (week before procedure), immediate post-procedure, 6 weeks, and up to 6 months post-procedure (Figure 2). No participants were lost to follow-up.

To reduce inter-case variability and to support consistent documentation, outcomes were assessed using a standardized follow-up schedule and a standardized video protocol (same patient positioning, maneuver sequence, and camera viewpoint when feasible).

Primary (feasibility) qualitative outcomes were: (1) technical completion of planned targets; (2) tolerability under local anesthesia (including pain during the procedure); (3) adverse events and serious adverse events; and (4) completion of planned follow-up. Dysesthesia, new neuropathic pain, skin injury, infection, hematoma, and motor deficit beyond expected were the adverse events that were actively looked for.

Secondary outcomes included spasticity clinical assessment with MAS and standardized video recordings before/during/after the procedure to document changes in posture and passive mobility. Passive mobility was assessed qualitatively during standardized clinical handling and documented by video recordings. The examiner evaluated the ease of passive mobilization and resistance to passive movement during passive elbow extension, forearm supination, wrist extension, and passive finger opening in the spastic upper limb.

Videos were used to document observable changes in posture and the ease of passive mobilization; they were not used to quantify joint angles. No goniometric measurements were performed. Therefore, results are reported as changes in muscle tone using the Modified Ashworth Scale and as qualitative changes in passive sequential joint mobility, rather than as measurements of passive range of motion. Video capture has been used in previous CNL reports in spasticity to document pre- and post-intervention clinical examination findings and to illustrate both technique and outcomes [66].

Feasibility endpoints were assessed descriptively and were considered met when all planned targets were treated, follow-up was completed, no serious adverse events occurred, and the procedure was tolerated under local anesthesia.

### 2.6. Pain and Sensory Testing

Pain and sensory testing were performed by the same occupational therapist 2 weeks before and 6 weeks after CNL. Neuropathic pain screening used the DN4 questionnaire [77]. Pain intensity was recorded on a visual analogue scale (VAS). Sensory testing in the distribution of the lateral antebrachial cutaneous nerve (terminal sensory branch of the musculocutaneous nerve) included pressure perception threshold, static two-point discrimination, and thermal sensation.

### 2.7. Electroneuromyography

To document peripheral nerve integrity and electrophysiological changes, ENMG was performed 1 week before and 6 weeks after the intervention by an experienced physician. Nerve conduction studies included motor and sensory studies (action potential amplitude, conduction velocity, and F-wave latency) for the median and ulnar nerves. Needle EMG examined the biceps brachii, abductor digiti minimi, first dorsal interosseous, and abductor pollicis muscles, and was specifically used to assess the presence of spontaneous activity (fibrillation potentials and positive sharp waves) in muscles innervated by the cryoneurolyzed nerve, to detect possible post-procedural denervation changes.

### 2.8. Statistical Analysis

Given the small sample size and feasibility design, analyses were descriptive.

## 3. Results

### 3.1. Participant Characteristics and Targets

Three chronic post-stroke hemiparetic outpatients (two men; age range 34–58 years) with a non-functional spastic upper limb were included (Table 1). Time from stroke to CNL ranged from 22 to 36 months. Two participants had a hemorrhagic stroke, and one had an ischemic stroke.

CNL targeted the musculocutaneous and median nerves in all participants; the ulnar nerve was additionally treated in participants #2 (Video S1, alternatively see video at: https://www.youtube.com/watch?v=uO8CGYXSLIE (accessed on 10 May 2021)) and #3 (Video S2, alternatively see video at: https://www.youtube.com/watch?v=IhLLSzIZLG0 (accessed on 26 June 2020)) (Table 2). Ulnar nerve CNL was not planned for participant #1 based on pre-procedure target selection.

### 3.2. Feasibility and Safety Observations

All planned CNL targets were treated as intended, and follow-up assessments were completed as scheduled. No procedure-related complications (e.g., infection, hematoma, skin injury, new neuropathic pain, or unexpected motor deficit) were observed during follow-up. Pain outcomes were unchanged or improved, and no clinically meaningful sensory loss was reported on bedside sensory testing. No clinically apparent autonomic/vegetative changes (temperature or sweating) were noted following the procedure.

### 3.3. Clinical Spasticity Outcomes (Tone and Joint Mobility)

All three participants demonstrated improvement in spasticity severity at the treated patterns, with a >2-point reduction in MAS for the targeted muscle groups (elbow flexors, forearm pronators, wrist and finger flexors) (Table 2).

**Figure 3:**
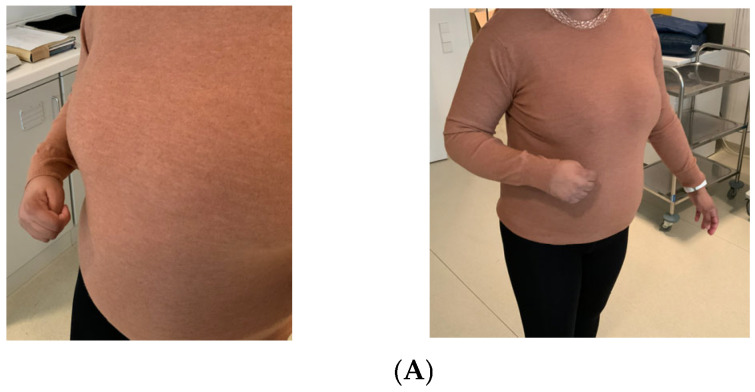

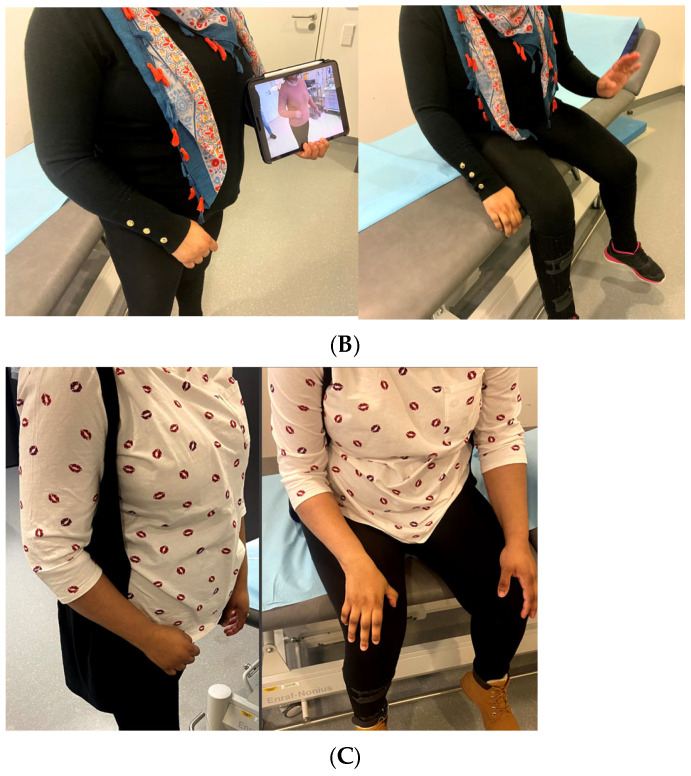
Effect of cryoneurolysis before (**A**), 3 months (**B**) and 6 months (**C**) after the cryoneurolysis of the musculocutaneous, median and ulnar nerves of patient #2.

**Figure 4:**
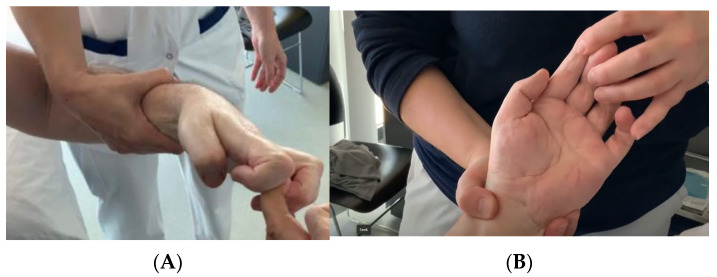
Left spastic wrist and hand before procedure for upper limb in patient #3 (**A**). Note that the hand is easily opened after the procedure (**B**).

The decrease in hypertonicity, changes in posture and improved passive mobility persisted for at least 6 months (Figure 3). Immediate post-procedure changes in wrist and hand posture and passive mobility were also visible in the clinical recordings (Figure 4).

### 3.4. Pain, Sensory Testing and Ease of Care

DN4 remained unchanged in participants #1 and #3 (0 → 0) and decreased in participant #2 (6 → 5). VAS was unchanged in participants #1 and #3 (0 → 0) and improved in participant #2 (5.5 → 0).

Sensory testing showed preserved thermal sensation before and after CNL in participants #1 and #2; participant #3 had absent two-point discrimination and impaired hot temperature perception both before and after. Pressure threshold decreased in participant #1 (1 g → 0.4 g) and was unchanged in participants #2 and #3 (0.7 g → 0.7 g). Two-point discrimination was unchanged in participants #1 and #2.

All participants reported improved ease of passive care (e.g., easier hand opening and hygiene handling) after the procedure; however, this outcome was assessed qualitatively and not measured using a standardized tool. The use of validated instruments, such as the Caregiver Burden Scale, would be important in future studies to more objectively capture this dimension. No participant reported new unpleasant sensory symptoms at follow-up.

### 3.5. Electroneuromyography Findings

Electrophysiological findings were heterogeneous (Table 2). Participant #1 showed no change in median nerve motor amplitudes or conduction studies, which were normal at baseline, and no spontaneous denervation activity on post-intervention needle EMG. Participant #2 had no change in median and ulnar motor/sensory amplitudes, conduction velocities, or F-wave latency (baseline normal), although post-intervention EMG interpretation was limited by spasms. Participant #3 had stable median nerve conduction parameters and normal EMG in the first dorsal interosseous and abductor pollicis brevis; however, the ulnar sensory action potential amplitude decreased from ~30% pre-intervention to ~60% post-intervention, with preserved F-wave latency, and biceps brachii EMG showed spontaneous denervation activity (fibrillation potentials and positive sharp waves) after CNL (Table 2).

## 4. Discussion

This feasibility case series examined whether ultrasound- and stimulation-guided CNL targeting upper-limb peripheral nerves can be delivered in an outpatient rehabilitation setting and whether a multidimensional follow-up (clinical and neurophysiological) can be completed. All planned procedures were completed as intended, and follow-up data were obtained, supporting procedural feasibility within this care pathway. Clinically, all participants demonstrated a reduction in hypertonicity with improved qualitatively observed passive mobility (video-documented) in the targeted patterns, and these changes were observed immediately after the intervention and persisted through the 6-month clinical follow-up.

Importantly, passive mobility outcomes in this study were qualitatively observed rather than quantified by goniometry or Modified Tardieu angles. The clinically relevant construct was the ease of passive mobilization (e.g., ease of elbow extension, forearm supination, wrist/finger opening) during routine handling, which is directly aligned with common patient- and caregiver-centred goals in a non-functional spastic upper limb (comfort, hygiene, dressing, positioning, and prevention of secondary complications). This distinction should be considered when interpreting “mobility” in the present case series and when designing future studies that aim to quantify angular changes.

From a mechanistic perspective, CNL is generally considered to induce an axonotmesis-type lesion while preserving the connective-tissue scaffolding of the nerve, which may facilitate regeneration and functional recovery [55,56]. This theoretical reversibility is one reason CNL is attractive as an alternative to more destructive procedures in selected cases. In contrast, chemical neurolysis with phenol or alcohol has a long-standing role in spasticity management but may be associated with neuritis/dysesthesia and local tissue effects, and risk–benefit considerations can be more complex when treating mixed sensorimotor nerves [35,36,37,38,39]. Accordingly, CNL may be clinically appealing when mixed nerves are relevant targets, if patients are counselled regarding the possibility of sensory change and that monitoring is systematic.

Our findings also support considering CNL as part of goal-oriented, multimodal spasticity management rather than as a stand-alone intervention. Because its effect is observable immediately after treatment, CNL enables a stepwise approach across sequential sessions. Subsequent sessions may then include interventions with a slower onset of action, such as botulinum toxin injections [28,29,32]. In principle, CNL could also be used to address broader spasticity patterns (e.g., through mixed nerves or major motor branches), potentially allowing botulinum toxin dosing to be focused on individual muscles that are difficult to access with CNL or where fine-tuning and toxin-sparing strategies are desirable.

However, the clinical value of combined sessions, optimal sequencing, and the incremental benefit over usual care remains unknown and requires evaluation in controlled studies [78].

CNL represents an innovative therapeutic option for patients with spasticity who have reached a plateau in their functional response to conventional treatments [71]. By reducing spastic overactivity, CNL may also optimize the use of functional electrical stimulation (FES) neuroorthoses and enhance their functional effectiveness [74].

Compared with surgical options such as peripheral nerve neurotomy, CNL is percutaneous and can be performed under local anesthesia, potentially reducing procedural burden and peri-procedural risk [58,66]. At the same time, wider implementation may be limited by equipment costs, the need for training in peripheral nerve sonoanatomy and ultrasound-guided interventions, and the currently limited evidence base in spasticity relative to interventional pain practice. These constraints highlight the importance of pragmatic feasibility reporting and shared protocols to standardize target selection and dosing parameters.

Safety interpretation deserves particular attention. Clinically, no new sensory deficit was detected on bedside testing at 6 weeks. However, ENMG findings were heterogeneous and included changes in one participant that warrant cautious interpretation (e.g., further reduction in ulnar sensory action potential amplitude and denervation activity in the biceps). Importantly, CNL is known to induce a reversible axonotmesis through the Wallerian degeneration distal to the lesion while preserving the connective-tissue architecture of the nerve. Therefore, the presence of denervation activity on needle EMG may represent an expected electrophysiological consequence of the mechanism of action of the technique rather than a pathological complication.

Although the small sample size precludes any inference about frequency, these observations support the need for systematic neurophysiological monitoring in future studies, particularly when mixed nerves are targeted. They also suggest that future protocols should explore whether targeting more distal motor branches or intramuscular nerve branches can improve the balance between motor benefit and sensory/autonomic safety. In addition to standardized adverse-event reporting, future protocols could explicitly compare mixed-nerve targeting versus more distal motor-branch targeting to test whether motor benefit can be retained while reducing sensory risk signals.

Beyond feasibility in a single centre, implementation will depend on local workflow and expertise. The technique requires training in ultrasound-guided peripheral nerve identification and stimulation mapping and may be most effective when embedded in a structured post-procedure rehabilitation programme to consolidate newly available movement patterns. Procedural costs vary across health systems, and formal cost-effectiveness analyses are not yet available; these considerations should be incorporated into future pragmatic evaluations.

Strengths of this case series include standardized targeting confirmed by diagnostic nerve blocks, the use of ultrasound and stimulation to support procedural reproducibility, and multimodal follow-up, including clinical outcomes, sensory testing, and ENMG. However, several limitations should be acknowledged. First, this feasibility case series was not designed to establish efficacy and included only three participants; results may not generalize across etiologies, spasticity patterns, or levels of residual voluntary function. Second, outcomes were largely descriptive and clinically oriented; more standardized quantification of passive stiffness/biomechanics and patient-centred endpoints (comfort, ease of care, caregiver burden, participation) would strengthen future trials. Third, although immediate changes were observed, durability and optimal re-treatment intervals remain uncertain and may depend on freezing parameters (cycle duration, number of cycles), nerve characteristics, and the balance between neural and structural contributors to resistance.

Future studies should combine feasibility endpoints with a minimal, reproducible outcomes set: Modified Tardieu Scale angles and/or goniometry for passive joint excursion, goal attainment (e.g., hygiene/dressing/positioning goals), caregiver burden measures, and patient-centred outcomes. Where feasible, objective markers of re-innervation and function (e.g., dynamometry and structured electrophysiology) may help characterize dose–response relationships and durability, while instrumented movement analysis can strengthen causal attribution in selected subgroups.

## Figures and Tables

**Table 1:** General characteristics of the participants included in the study.

Patient	Sex	Age (Years)	Type of Stroke	Affected Side	Time Delay Between Stroke and CNL (Months)
#1	man	45	hemorrhagic	right	25
#2	woman	34	hemorrhagic	right	36
#3	man	58	ischemic	left	22

CNL: Cryoneurolysis.

**Table 2:** Targeted nerves and assessment results for participants.

Participant	Nerves Treated	Neuropathic Pain (DN4) (Pre → 6 Wks)	Pain VAS (Pre → 6 Wks)	Sensory Testing (6 Wks) Pressure (g); 2-Point (mm); Thermal	Spasticity/Mobility	ENMG (6 Wks) Median	ENMG (6 Wks) Ulnar/Musculocutaneous
#1	Musculocutaneous; median	0 → 0	0 → 0	1.0 → 0.4 g; 48 → 48 mm; thermal preserved	MAS ↓ > 2 points (elbow flexors, pronators, wrist/finger flexors); passive mobility improved (no video)	No change (baseline normal); no EMG rest activity post	Ulnar: not treated/not assessed Musculocutaneous: no denervation reported
#2	Musculocutaneous; median; ulnar	6 → 5	5.5 → 0	0.7 → 0.7 g; 25 → 25 mm; thermal preserved	MAS ↓ > 2 points; passive mobility improved (Video S1)	No change (baseline normal)	Ulnar: no change (baseline normal) Needle EMG: interpretation limited by spasms (rest activity not excluded)
#3	Musculocutaneous; median; ulnar	0 → 0	0 → 0	0.7 → 0.7 g; no 2-point discrimination pre/post; hot sensation absent pre/post	MAS ↓ > 2 points; passive mobility improved (Video S2)	No change (baseline normal); EMG FDI/APB normal	Ulnar SNAP amplitude reduction progressed (~30% → ~60%); F-wave latency normal; ADM EMG normal Musculocutaneous: biceps EMG showed denervation activity post

DN4, Douleur Neuropathique 4 questionnaire; VAS, visual analogue scale; MAS, Modified Ashworth Scale; ENMG, electroneuromyography; SNAP, sensory nerve action potential; FDI, first dorsal interosseous; APB, abductor pollicis brevis; ADM, abductor digiti minimi. ↓ indicates a decrease.

## Data Availability

The original contributions presented in this study are included in the article and Supplementary Material including with the de-identified videos supporting the findings. Further inquiries, especially any additional de-identified materials can be directed to the corresponding author.

## Supplementary Materials

The following supporting information can be downloaded at: https://www.mdpi.com/article/10.3390/neurolint18050078/s1, Video S1: Cryoneurolysis to treat upper limb spasticity pilot study patient N2; Video S2: Cryoneurolysis to treat upper limb spasticity pilot study patient N3.
